# Route-Specific Meningo-Ophthalmic and Orbitomeningeal Communications Relevant to Middle Meningeal Artery Embolization: A Systematic Review and Meta-Analysis

**DOI:** 10.3390/neurolint18070128

**Published:** 2026-07-06

**Authors:** Alejandro Bruna-Mejias, Loreto Paez-Allendes, Valentina Perez-Lira, Diego Santander-Chavez, Mathis Miranda-Schoen, Juan José Valenzuela-Fuenzalida, María P. Moya, Gustavo Oyanedel-Amaro, Gloria Cifuentes-Suazo, Mathias Orellana-Donoso, Juan J. Cabezas-Salgado, Cristopher Blackwood-Espinoza, Juan Sanchis-Gimeno

**Affiliations:** 1 Departamento de Ciencias y Geografía, Facultad de Ciencias Naturales y Exactas, Universidad de Playa Ancha, Valparaíso 2360072, Chile; loreto.paez@upla.cl; 2 Escuela de Medicina, Facultad de Medicina, Universidad Andrés Bello, Viña del Mar 2520000, Chilejuan.kine.2015@gmail.com (J.J.V.-F.); 3 Departamento de Ciencias Química y Biológicas, Facultad de Ciencias de la Salud, Universidad Bernardo O’Higgins, Santiago 8370993, Chile; 4 Facultad de Ciencias de la Salud, Universidad Autónoma de Chile, Santiago 8910060, Chile; 5 Faculty of Health and Social Sciences, University of the Americas, Santiago 8370040, Chile; 6 Facultad de Medicina, Carrera de Odontología, Universidad Católica de la Santísima Concepción, Av. Alonso de Ribera 2850, Concepción 4090541, Chile; 7 Escuela de Medicina, Universidad Finis Terrae, Santiago 7501015, Chile; 8 Facultad de Medicina y Ciencia, Universidad San Sebastián, Lota 2465, Santiago 7510157, Chile; 9 Facultad de Medicina, Universidad Católica del Maule, Talca 3460000, Chile; 10 Escuela de Ciencias de la Salud, Universidad Viña del Mar, Viña del Mar 2520000, Chile; 11 GIAVAL Research Group, Department of Anatomy and Human Embryology, Faculty of Medicine, University of Valencia, 46001 Valencia, Spain; juan.sanchis@uv.es

**Keywords:** middle meningeal artery, ophthalmic artery, meningolacrimal artery, meningo-ophthalmic anastomosis, embolization, chronic subdural hematoma, prevalence, systematic review, meta-analysis

## Abstract

Purpose: Meningo-ophthalmic and orbitomeningeal arterial communications comprise route-specific relationships between the middle meningeal artery (MMA) and the ophthalmic or orbital arterial system. Their recognition is relevant to middle meningeal artery embolization because orbital or ophthalmic collateral pathways may create routes for non-target embolization. This systematic review aimed to synthesize the prevalence and anatomical patterns of these communications, using quantitative pooling only where the anatomical definition and denominator were sufficiently coherent. Methods: This systematic review and meta-analysis were conducted according to PRISMA 2020 principles and registered in PROSPERO (CRD420261361050). Eligible studies were original human cadaveric anatomical, angiographic, or radiological investigations reporting MMA-ophthalmic or MMA-orbital arterial relationships. After the closure of the full-text retrieval audit, studies and extracted rows were audited by anatomical family, unit of analysis, numerator, denominator, and independence. No global pooled prevalence was calculated across anatomical families. When family-specific pooling was methodologically defensible, proportions were synthesized using logit transformation, restricted maximum likelihood random-effects models, and Hartung-Knapp confidence intervals. Results: Database searches identified 558 records. After removal of 228 duplicates, 330 records were screened, and 285 were excluded by title and abstract. Forty-five reports were sought for retrieval; 10 were not retrieved or were not available as assessable full-text reports after retrieval auditing. Thirty-five full-text reports were assessed; thirteen were excluded for reasons, and three were duplicate reports at the full-text stage. Nineteen studies were included in the qualitative synthesis, and 12 contributed independent data to the final R-ready matrix. MMA arising from the ophthalmic artery was uncommon, with a pooled prevalence of 0.03 (95% CI 0.01 to 0.13; I^2^ = 75.9%). After excluding the clinically selected chronic subdural hematoma subgroup, the estimate was 0.02 (95% CI 0.01 to 0.06; I^2^ = 7.7%). The meningolacrimal/lacrimal-MMA route yielded an exploratory pooled proportion of 0.45 (95% CI 0.09 to 0.86; I^2^ = 95.9%), with substantial anatomical and methodological heterogeneity. Conclusions: The available evidence supports route-specific synthesis rather than a single global prevalence estimate. MMA arising from the ophthalmic artery appears uncommon but procedurally important; however, this estimate should be interpreted as a route-specific estimate across eligible angiographic/anatomical series rather than as a universal anatomical prevalence. Meningolacrimal and lacrimal-MMA routes are frequently described, but their prevalence remains difficult to generalize because detection methods, populations, and denominators differ across studies. Future anatomical and angiographic reports should standardize route definitions, laterality, unit of analysis, and denominator reporting to improve prevalence estimation and procedural safety interpretation.

## 1. Introduction

The middle meningeal artery (MMA) is usually described as the dominant dural branch of the maxillary artery within the external carotid arterial system, whereas the ophthalmic artery (OA) is classically supplied by the internal carotid artery and reaches the orbit through the optic canal. This scheme remains useful for teaching and procedural orientation, but it is anatomically incomplete. Full-text review of the classical angiographic and microsurgical literature confirms that MMA branches may communicate with ophthalmic or orbital territories through pathways that cross the expected boundary between external carotid, internal carotid, lacrimal, orbital, and dural circulations [1,2,3].

These relationships are best understood as route-specific patterns rather than as a single meningo-ophthalmic variant. The reported configurations include MMA arising from the OA, OA arising from the MMA, direct OA-MMA anastomoses, meningolacrimal or lacrimal-MMA routes, recurrent meningeal routes, sphenoidal routes, and anterior falx-related collateral pathways [1,2,4,5,6,7,8,9,10]. Some of these patterns represent anomalous arterial origins, whereas others correspond to collateral or route-specific communications between ophthalmic, lacrimal, orbital, and meningeal branches. Grouping them under a single label may be convenient, but it risks obscuring clinically meaningful differences in direction of flow, route of entry into the orbit, target territory, and angiographic detectability.

A concise developmental perspective also supports this family-based approach. The orbital and meningeal arterial systems are related during cranial vascular development through primitive channels that normally regress, remodel, or become incorporated into the definitive carotid systems. Differential persistence or regression of these channels can account for an OA supplied through the MMA, an MMA arising from the ophthalmic system, or smaller orbitomeningeal communications through lacrimal, recurrent meningeal, sphenoidal, or falx-related routes [2,4,5,6,7,8,10]. These mechanisms do not imply that all routes are functionally equivalent in adult life; rather, they explain why similar territories may remain connected through different anatomical pathways.

The contemporary relevance of these variants has increased with the expanding use of MMA embolization for chronic or nonacute subdural hematoma. Recent angiographic series and procedural imaging studies show that the MMA may have variant origins or ophthalmic/orbital connections that are relevant to treatment planning, feasibility, and safety [11,12,13,14,15]. In selected configurations, embolic material delivered through the MMA system may have access to orbital or ophthalmic territories if the route is not recognized before or during treatment. This possibility is not limited to a single named vessel. A proximal anomalous origin has different implications from a distal meningolacrimal, recurrent meningeal, or sphenoidal route, and angiographic non-visualization does not necessarily exclude a small or competitively filled communication.

The same anatomical region has been examined through markedly different methods, including injected cadaveric material, microsurgical dissection, selective and superselective angiography, three-dimensional rotational angiography, magnetic resonance angiography, and CT-supported procedural assessment [1,2,3,4,5,6,7,8,9,10,11,12,13,14,15,16,17,18,19]. These methods do not capture identical phenomena. Cadaveric studies may reveal vessels that are not opacified under in vivo flow conditions, whereas clinical angiography depends on injection selectivity, field of view, competitive flow, and the indication for imaging. As a result, prevalence estimates derived from individual studies are strongly influenced by the anatomical definition, detection method, and unit of analysis used.

The present systematic review and meta-analysis were therefore designed to synthesize MMA-ophthalmic and MMA-orbital relationships by anatomical family. The aim was to estimate pooled prevalence only where the anatomical definition, numerator, denominator, and independence of observations were sufficiently coherent, and to summarize non-poolable evidence through structured qualitative synthesis. This route-specific approach was chosen to preserve anatomical meaning while still extracting quantitative information where the evidence allowed it.

## 2. Materials and Methods

### 2.1. Protocol and Registration

This systematic review and meta-analysis was conducted in accordance with PRISMA 2020 principles [20]. The completed PRISMA 2020 checklist is provided as Supplementary Table S7. The protocol was registered in PROSPERO (CRD420261361050). A protocol amendment was made to clarify the handling of selected pediatric angiographic series and mixed-age or cadaveric anatomical datasets that provided direct evidence on MMA-ophthalmic or MMA-orbital arterial pathways. These data were retained for anatomical mapping and, where appropriate, exploratory synthesis; they were not interpreted as estimates of adult population prevalence. The amendment text is provided in the Supplementary Material.

### 2.2. Eligibility Criteria

Eligible studies were original human anatomical, angiographic, or radiological investigations that reported arterial communications, anomalous origins, or route-specific relationships involving the middle meningeal artery and the ophthalmic or orbital arterial system. Eligible evidence included cadaveric dissection, cadaveric angiography, digital subtraction angiography, computed tomography angiography, magnetic resonance angiography, 3D rotational angiography, and related imaging methods, provided that the relevant vascular pathway could be identified and the anatomical finding could be extracted. Studies were eligible for quantitative synthesis only when an event count, a denominator, a unit of analysis, and an anatomical family could be defined without duplicating or nesting data.

Reports were excluded when they did not provide original data, were narrative reviews, commentaries, letters, image-only reports, or single illustrative cases without an eligible denominator, had the wrong anatomical focus, described only clinical complications without documenting the anatomical or angiographic pathway, or represented duplicate reports of the same data. Studies with clinically selected or non-adult samples were not automatically excluded if they provided relevant anatomical evidence, but their role was restricted according to the final poolability audit.

### 2.3. Electronic Search

Searches were performed in PubMed, Scopus, Web of Science, CINAHL, and LILACS. No publication-year limits were applied in the recorded strategies, and no automated language, human-participant, or document-type filters were applied in the database search strings available for audit. The strategies combined terms for the middle meningeal artery, ophthalmic or orbital arteries, meningolacrimal and related orbital routes, and anastomotic, collateral, variant, origin, or branching patterns. The complete database-specific strategies, limits or notes, verified source yields, and search-date fields are provided in Supplementary Table S1.

### 2.4. Study Selection

Records were deduplicated before title and abstract screening. Potentially eligible records were sought for full-text retrieval, and full-text reports were assessed against the predefined eligibility criteria. Reports that could not be retrieved, or that were not available as assessable full-text reports after retrieval auditing, were counted as reports not retrieved in the PRISMA flow and were not entered into the eligibility denominator. Reasons for exclusion at the full-text stage were standardized and recorded. Duplicate reports identified during eligibility assessment were counted separately from exclusions and were not allowed to contribute duplicate data. The final retrieval and eligibility set was closed before the definitive poolability audit and quantitative synthesis. Full-text exclusions and duplicate reports identified during eligibility assessment are detailed in Supplementary Tables S2 and S2a.

### 2.5. Data Collection Process

Data were extracted into structured matrices designed for prevalence and anatomical-route synthesis. Extracted variables included author and year, country or setting when reported, study material or population, assessment method, sample size, unit of analysis, anatomical focus, route or direction of communication, numerator, denominator, and notes relevant to independence or overlap. When a study reported multiple related events, the extracted rows were audited to determine whether they represented independent observations, mutually exclusive subcategories, conditional denominators, or nested descriptions of the same anatomical finding. The final independent-row quantitative matrix and the rows dropped or merged during the poolability audit are provided in Supplementary Tables S4 and S5.

### 2.6. Anatomical Family Classification and Poolability Audit

A single global pooled prevalence was not calculated because the included studies did not measure one interchangeable anatomical entity. A global estimate would have combined anomalous arterial origins, direct anastomoses, lacrimal or recurrent routes, sphenoidal routes, and conditional subseries with different denominators and procedural implications. Instead, extracted events were classified into predefined anatomical families: MMA from OA, OA from MMA, direct OA-MMA anastomosis, meningolacrimal/lacrimal-MMA route, recurrent meningeal route, sphenoidal route, and anterior falx route. The final quantitative matrix retained only independent rows after removing duplicate, nested, conditional, or overlapping events. Laterality-specific rows were not treated as independent when they were nested within a global patient-level or any-side denominator; in those cases, the most appropriate global event was retained for descriptive or quantitative use, and the laterality rows were excluded from the R input. Poolability was judged at the study-family level according to anatomical definition, unit of analysis, denominator compatibility, event independence, and method of detection. This audit was an anatomical and epidemiological safeguard rather than a purely statistical step: it was designed to prevent double-counting, nested denominators, conditional subseries, overlapping route definitions, and artificial inflation of precision.

### 2.7. Assessment of the Methodological Quality of the Included Studies

Methodological quality was assessed using a JBI-informed framework for prevalence evidence [21]. The appraisal considered the sampling frame, sampling method, sample size, description of subjects or anatomical material, coverage of the target population or material, validity and consistency of the identification method, adequacy of the analysis, and completeness of reporting. Because anatomical evidence may come from cadaveric material, historical series, or selected angiographic samples, the appraisal was not used as a mechanical exclusion rule. Instead, it informed the interpretation and synthesis role assigned to each study.

Because this review synthesized anatomical prevalence and route-specific descriptive evidence rather than intervention effects or comparative clinical outcomes, a formal GRADE assessment was not applied. Certainty and interpretability were instead addressed through JBI-informed methodological appraisal, route-specific poolability assessment, denominator compatibility, independence of observations, heterogeneity metrics, prediction intervals when applicable, and synthesis-tier assignment.

### 2.8. Statistical Methods

No global pooled prevalence was calculated across all meningo-ophthalmic or orbitomeningeal pathways. This decision was made before the final interpretation of the pooled estimates because the routes differed in direction of flow, anatomical definition, unit of analysis, denominator, and clinical meaning. Quantitative synthesis was therefore restricted to anatomically coherent families after the row-level poolability audit. For eligible family-specific analyses, proportions were transformed using the logit transformation. Random-effects models were fitted using restricted maximum likelihood, and Hartung-Knapp confidence intervals were used for pooled estimates [22,23]. Individual study confidence intervals were calculated as exact binomial intervals. Prediction intervals were reported when applicable. Heterogeneity was described using I^2^, tau^2^, and the corresponding heterogeneity test. Statistical analyses were performed in RStudio version 2026.04.0+526 using R version 4.5.2 and the metafor package version 5.0-1. Family-specific random-effects meta-analyses of proportions were fitted using restricted maximum likelihood estimation with Hartung-Knapp confidence intervals.

### 2.9. Sensitivity and Subgroup Analyses

A sensitivity analysis was conducted for MMA arising from the ophthalmic artery after excluding the clinically selected chronic subdural hematoma subgroup reported by Fantoni et al. For the meningolacrimal/lacrimal-MMA route, an exploratory subgroup analysis separated cadaveric/anatomic evidence from imaging/angiographic evidence. These analyses were interpreted descriptively and were not treated as confirmatory tests of subgroup differences, given the small number of contributing studies and the anatomical heterogeneity of the evidence.

## 3. Results

### 3.1. Included Articles

**Figure 1:**
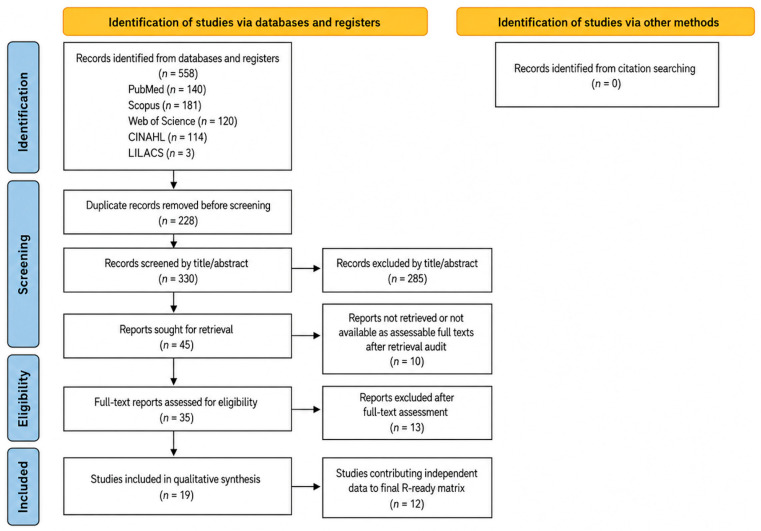
PRISMA 2020 flow diagram. Final flow after full-text retrieval auditing.

The database searches identified 558 records: PubMed (*n* = 140), Scopus (*n* = 181), Web of Science (*n* = 120), CINAHL (*n* = 114), and LILACS (*n* = 3). No additional records were added through citation searching. After removal of 228 duplicates, 330 records were screened by title and abstract, and 285 were excluded. Forty-five reports were sought for retrieval; 10 could not be retrieved or were not available as assessable full-text reports after retrieval auditing. Thirty-five full-text reports were assessed for eligibility. Thirteen reports were excluded for various reasons, and three duplicate reports were identified at the full-text stage. Nineteen studies were included in the qualitative synthesis, of which 12 contributed independent data to the final quantitative matrix. The study selection process is summarized in Figure 1.

### 3.2. Characteristics of the Studies and the Study Population

The final qualitative synthesis comprised 19 studies published between 1974 and 2025 [1,2,3,4,5,6,7,8,9,10,11,12,13,14,15,16,17,18,19]. The evidence included cadaveric anatomical dissection, injected cadaveric material, cadaveric angiography, DSA, 3D rotational angiography, MRA, CT/CTA-supported angiographic assessment, and preprocedural imaging review. The included reports did not represent a single uniform population denominator. Instead, they contributed route-specific anatomical information with different units of analysis, including patients, arteries, hemispheres, orbits, dissections, angiographies, and MMA specimens. This heterogeneity was the main reason for separating the evidence into anatomical families before any quantitative synthesis. The main characteristics of the studies included in the qualitative synthesis are summarized in Table 1.

### 3.3. Description of Variants and Poolability Decisions

The family-level audit confirmed that MMA-ophthalmic and/or bitomeningeal communications should not be treated as one anatomical variant. The included studies described several directionally and morphologically distinct patterns: MMA arising from the OA, OA arising from the MMA, direct OA-MMA anastomosis, meningolacrimal or lacrimal-MMA routes, recurrent meningeal routes, sphenoidal routes, and anterior falx-related collateral pathways [1,2,3,4,5,6,7,8,9,10,11,12,13,14,15,16,17,18,19]. Only families with ex-tractable, non-overlapping, and sufficiently comparable numerators and denominators were taken forward to quantitative synthesis. Families with sparse evidence, conditional denominators, or incompatible definitions were retained for descriptive anatomical synthesis. The decision to retain, merge, exclude, or downgrade extracted rows was therefore based on whether each row represented an independent anatomical observation with a compatible denominator, not merely on whether a numerical event count was available. For readers less familiar with this anatomical literature, the operational route definitions used in this review are summarized in Table 2. Family-specific poolability decisions are shown in Table 3.

### 3.4. Prevalence

**Figure 2:**
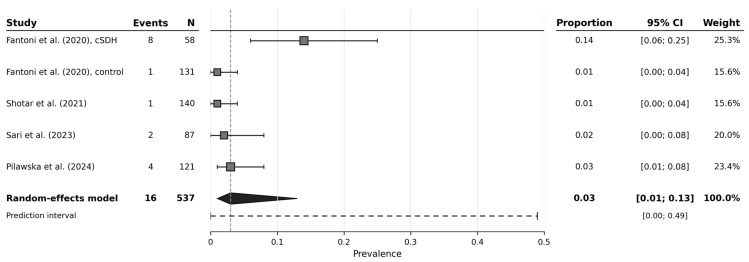
Prevalence of the middle meningeal artery arising from the ophthalmic artery. The forest plot includes Fantoni et al. (cSDH and control subgroups), Shotar et al., Sari et al., and Pilawska et al. [11,12,13,14]. Family-specific random-effects meta-analysis was performed using logit-transformed proportions with restricted maximum likelihood estimation and Hartung-Knapp confidence intervals. Squares represent study- or subgroup-specific proportions, with square size proportional to the random-effects weight; horizontal lines indicate exact binomial 95% confidence intervals. The bolded row and diamond represent the pooled random-effects estimate, and the dashed horizontal line represents the prediction interval. No global pooled prevalence across anatomical families was performed.

For MMA arising from the ophthalmic artery, five independent rows from four studies or subgroups contributed 16 events among 537 evaluated units [11,12,13,14]. The pooled prevalence was 0.03 (95% CI 0.01 to 0.13). The prediction interval ranged from 0.00 to 0.49, and between-study heterogeneity was substantial (I^2^ = 75.9%, tau^2^ = 1.2534, *p* = 0.001). This was the most methodologically defensible primary quantitative estimate because all contributing rows described the same directional variant. Figure 2 shows the family-specific forest plot for MMA arising from the ophthalmic artery.

**Figure 3:**
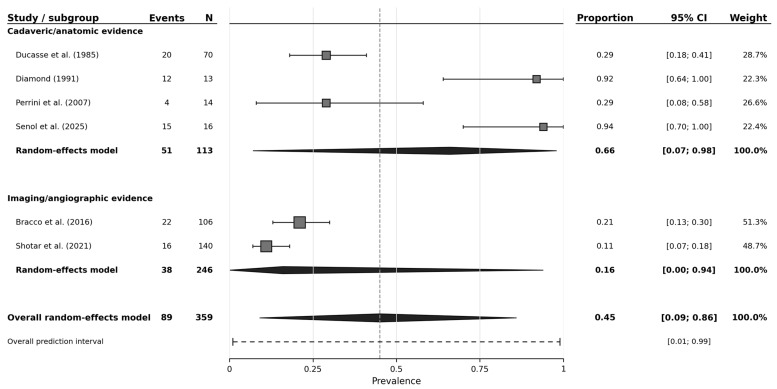
Meningolacrimal/lacrimal-MMA route by evidence source. The forest plot includes cadaveric/anatomic evidence from Ducasse et al., Diamond, Perrini et al., and Senol et al., and imaging/angiographic evidence from Bracco et al. and Shotar et al. [3,4,8,12,18,19]. Proportions were synthesized using logit transformation, restricted maximum likelihood random-effects models, and Hartung-Knapp confidence intervals. Squares represent study-specific proportions, with square size proportional to the random-effects weight; horizontal lines indicate exact binomial 95% confidence intervals. The bolded subgroup rows and diamonds represent subgroup random-effects estimates, whereas the bolded overall row and diamond represent the overall random-effects estimate. The dashed horizontal line represents the overall prediction interval. Subgroup estimates are descriptive and should not be interpreted as confirmatory tests of subgroup differences because of the small number of contributing studies, wide confidence intervals, and substantial anatomical and methodological heterogeneity. Original figure created by the authors from the data extracted for this systematic review; no third-party material was reproduced or adapted.

**Figure 4:**
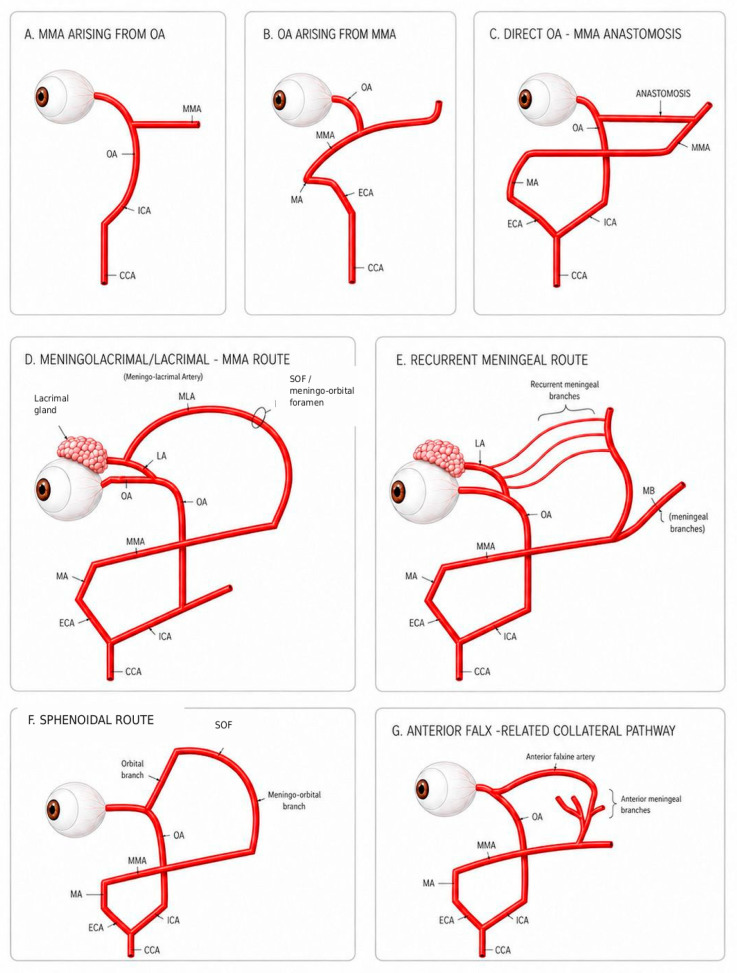
Schematic representation of major route-specific meningo-ophthalmic and orbitomeningeal communications. Panels A-G illustrate the principal route families considered in this review: (**A**) MMA arising from OA, (**B**) OA arising from MMA, (**C**) direct OA-MMA anastomosis, (**D**) meningolacrimal/lacrimal-MMA route, (**E**) recurrent meningeal route, (**F**) sphenoidal route, and (**G**) anterior falx-related collateral pathway. The figure is schematic and intended to clarify route definitions rather than depict all possible anatomical variants. Abbreviations: CCA, common carotid artery; ECA, external carotid artery; ICA, internal carotid artery; LA, lacrimal artery; MA, maxillary artery; MB, meningeal branches; MLA, meningolacrimal artery; MMA, middle meningeal artery; OA, ophthalmic artery; SOF, superior orbital fissure.

For the meningolacrimal/lacrimal-MMA route, six studies contributed 89 events among 359 evaluated units [3,4,8,12,18,19]. The pooled proportion was 0.45 (95% CI 0.09 to 0.86), with a prediction interval from 0.01 to 0.99. Heterogeneity was considerable (I^2^ = 95.9%, tau^2^ = 3.1949, *p* < 0.001). This estimate was therefore retained as exploratory and supplementary rather than interpreted as a stable population prevalence. The wide confidence and prediction intervals reflect the marked differences between anatomical/cadaveric and imaging/angiographic evidence sources. The overall exploratory forest plot for the meningolacrimal/lacrimal-MMA route is provided as Supplementary Figure S1. In the exploratory subgroup analysis, estimates differed numerically between cadaveric/anatomic evidence and imaging/angiographic evidence; however, the small number of studies and wide intervals precluded confirmatory interpretation of subgroup differences. The subgroup forest plot is shown in Figure 3. A schematic overview of the main route-specific anatomical patterns is provided in Figure 4.

The sensitivity analysis excluding the clinically selected cSDH subgroup from Fantoni et al. is summarized in Table 4 and shown in Supplementary Figure S2. The complete quantitative synthesis, sensitivity analysis, and descriptive subgroup results are provided in Supplementary Table S6.

### 3.5. Risk of Bias of Included Articles

The JBI-informed appraisal identified recurring concerns related to selected clinical populations, small anatomical samples, heterogeneous units of analysis, conditional subseries, and historical reports with incomplete denominator detail [21]. No study was excluded solely because of methodological-quality concerns. Instead, the appraisal informed whether each study was used for primary quantitative synthesis, exploratory/supplementary synthesis, or narrative anatomical interpretation. A summary of the JBI-informed methodological-quality appraisal is provided in Table 5. The detailed study-level and item-level appraisal is provided in Supplementary Table S3.

### 3.6. Clinical Considerations

From a clinical perspective, the main finding is not the absolute prevalence of a single variant, but the repeated demonstration that the MMA and the ophthalmic/orbital arterial systems may communicate through several distinct routes [1,2,3,4,5,6,7,8,9,10,11,12,13,14,15,16,17,18,19]. This distinction matters because a small, route-specific connection can be clinically more important than its frequency suggests, particularly when embolic material is delivered through the MMA territory. The available evidence therefore supports careful preprocedural assessment of the MMA origin, ophthalmic artery origin, lacrimal/meningolacrimal channels, recurrent meningeal branches, sphenoidal routes, and any visible falx-related collateral pathway when MMA embolization or skull-base/orbital procedures are planned [3,5,11,12,13,14,15,16,17,18,19].

These findings should not be read as evidence that every MMA-ophthalmic or orbitomeningeal communication represents an absolute contraindication to treatment. Rather, the clinical relevance depends on direction of flow, calibre, target territory, embolic agent, catheter position, and the degree to which the ophthalmic circulation is functionally recruited at the time of angiography [11,12,13,14,15,17,19]. A practical implication is that the absence of a visible anastomosis on routine angiography should be interpreted cautiously in the presence of competitive flow or very small orbital branches, especially when prior anatomical work demonstrates that some channels may be more frequently present than they are angiographically opacified [12,19].

## 4. Discussion

This systematic review shows that communications between the MMA and the ophthalmic/orbital arterial system do not constitute a single anatomical entity. The included literature describes directionally and morphologically different patterns, including MMA arising from the ophthalmic artery, ophthalmic artery arising from the MMA, direct OA-MMA anastomoses, meningolacrimal/lacrimal-MMA routes, recurrent meningeal routes, sphenoidal routes, and anterior falx-related collateral pathways [1,2,3,4,5,6,7,8,9,10,11,12,13,14,15,16,17,18,19]. In practical terms, pooling all these findings together would be equivalent to combining different anatomical questions under one denominator. This anatomical diversity explains why the most defensible synthesis strategy was not to calculate one global prevalence, but to separate the evidence into route-specific families and pool only those families with sufficiently coherent definitions and independent denominators.

The most methodologically defensible quantitative finding was the low pooled prevalence of MMA arising from the ophthalmic artery [11,12,13,14]. Although uncommon, this pattern is clinically important because the MMA may then be anatomically linked to the ophthalmic arterial axis rather than behaving as a conventional external carotid branch. The sensitivity analysis, excluding the chronic subdural hematoma subgroup from Fantoni et al., reduced heterogeneity substantially, indicating that selected clinical samples can influence the apparent frequency of this variant [11]. For that reason, the primary estimate should be interpreted as a family-specific prevalence across eligible angiographic series, not as a universal population estimate.

The meningolacrimal/lacrimal-MMA route required a different interpretation. Its pooled proportion was higher, but the confidence interval and prediction interval were wide, and heterogeneity was considerable [3,4,8,12,18,19]. This is anatomically plausible because cadaveric or cadaveric-angiographic studies may reveal channels that are not consistently visible in clinical angiography, while clinical series depend on flow conditions, injection technique, field of view, and the indication for imaging [3,12,18,19]. Therefore, this family is best presented as exploratory anatomical evidence rather than as a precise prevalence estimate.

Several families were deliberately not pooled despite their clinical interest. Ophthalmic artery arising from the MMA, sphenoidal routes, direct OA-MMA anastomoses, recurrent meningeal routes, and anterior falx-related pathways were retained for qualitative or exploratory interpretation when denominators were conditional, definitions differed, or the number of independent studies was too small [1,2,3,4,5,6,7,8,9,10,12,13,16,17,18]. This conservative decision avoids giving numerical precision to anatomical patterns that are currently better supported as descriptive or procedural-risk evidence.

The review also highlights a terminology problem that is relevant for both anatomy and intervention. Older anatomical papers often described meningo-orbital, sphenoidal, recurrent meningeal, or lacrimal connections according to dissection findings or foraminal relationships, whereas more recent angiographic studies tend to frame the same region in terms of embolization safety and dangerous collaterals [1,2,3,4,5,7,8,10,11,12,13,14,15,16,17,18,19]. These perspectives are complementary but not interchangeable. For future research, route-specific nomenclature, explicit directionality, laterality, unit of analysis, and denominator definition should be reported together.

Recent angiographic and procedural studies provide the contemporary clinical context for this anatomical synthesis. Fantoni et al. reported an ophthalmic origin of the MMA in patients evaluated for chronic subdural hematoma embolization, highlighting why the origin of the MMA should be checked before treatment [11]. Shotar et al. described the angiographic anatomy of the MMA in relation to chronic subdural hematoma embolization, including route-specific relationships with the ophthalmic system [12]. Sari et al. and Pilawska et al. further illustrate how three-dimensional rotational angiography and embolization-candidate series can identify MMA variants relevant to procedural planning [13,14], while Hubbard et al. emphasized the practical value of preprocedural CT assessment when MMA embolization feasibility is considered [15]. Together, these studies support the translational message of the present review: route recognition is more clinically useful than a single aggregate prevalence across heterogeneous pathways.

In relation to MMA embolization, the data support a cautious but not alarmist interpretation. The evidence does not allow estimation of the absolute risk of visual complications after embolization, because the included studies were not designed as complication-rate cohorts. What the review does show is that anatomical routes capable of linking the MMA territory to the orbital or ophthalmic territories are repeatedly documented, and that some are clinically silent until they become relevant during catheter-based procedures [3,5,11,12,13,14,15,16,17,18,19]. The safest translational message is therefore anatomical: embolization planning should include a deliberate assessment of these pathways, and future embolization studies should report whether and how they were screened.

### 4.1. Strengths

The main strength of this review is the row-level poolability audit performed before meta-analysis. Rather than treating all MMA-ophthalmic/orbital relationships as interchangeable, the evidence was separated into anatomical families, and nested, duplicate, conditional, or overlapping events were removed from the quantitative input. This approach is especially important in anatomical prevalence work, where the same study may describe several related structures using the same denominator. The review also combines historical anatomical evidence with contemporary angiographic evidence while preserving the methodological limits of each source [1,2,3,4,5,6,7,8,9,10,11,12,13,14,15,16,17,18,19].

### 4.2. Limitations

Several limitations should be acknowledged. First, the included studies differed in design, population, detection method, and unit of analysis [1,2,3,4,5,6,7,8,9,10,11,12,13,14,15,16,17,18,19]. Some were cadaveric anatomical studies, others were angiographic or radiological series, and selected clinical contexts included chronic subdural hematoma, retinoblastoma, or procedural feasibility assessment [11,12,14,15,18]. These differences limit the extent to which any pooled estimate can be interpreted as a general population prevalence.

Second, several anatomical families were supported by small samples, historical reports, or conditional denominators. This was particularly relevant for sphenoidal, recurrent meningeal, anterior falx, and direct OA-MMA pathways [1,2,3,4,5,6,7,8,9,10,12,13,16,17,18]. These data are clinically useful but cannot be forced into precise pooled estimates without risking overinterpretation.

Third, angiographic detectability is not equivalent to anatomical presence. Small vessels, competitive flow, selective injection technique, and imaging field may all affect whether a connection is seen [12,19]. Conversely, cadaveric demonstration of a vessel does not necessarily prove functional flow during a clinical procedure. This distinction explains why the quantitative results should be read alongside the qualitative anatomical synthesis rather than in isolation. Accordingly, the certainty and generalizability of route-specific prevalence estimates remain limited by heterogeneous denominators, selected clinical samples, modality-dependent detection, and the small number of independent studies in several anatomical families.

## 5. Conclusions

Meningo-ophthalmic and orbitomeningeal communications involving the middle meningeal artery and the ophthalmic/orbital arterial system are anatomically diverse and should not be summarized as a single global variant. In the available evidence, the most defensible quantitative estimate was for the middle meningeal artery arising from the ophthalmic artery, which appeared uncommon but clinically relevant for preprocedural assessment before middle meningeal artery embolization [11,12,13,14].

The meningolacrimal/lacrimal-MMA route was reported more frequently, particularly in anatomical and cadaveric-angiographic studies, but the pooled estimate was highly heterogeneous and should be interpreted as exploratory rather than as a stable population prevalence [3,4,8,12,18,19]. Other patterns, including ophthalmic artery arising from the MMA, direct OA-MMA anastomoses, recurrent meningeal, sphenoidal, and anterior falx-related routes, remain important for anatomical interpretation and procedural planning but currently require narrative or route-specific reporting rather than formal prevalence pooling [1,2,3,4,5,6,7,8,9,10,12,13,16,17,18].

Overall, the findings support careful route-specific angiographic evaluation before embolization procedures involving the MMA, explicit reporting of the direction and territory of any ophthalmic/orbital communication, and standardized denominators in future anatomical and clinical studies.

## Figures and Tables

**Table 1:** Characteristics of studies included in the qualitative synthesis.

Study	Year	Method	Main Anatomical Focus	Synthesis Role
McLennan et al. [1]	1974	Selective internal carotid angiography with radiologic-anatomic analysis	Ophthalmic-middle meningeal and stapedial-middle meningeal artery variants	Narrative anatomical synthesis
Lasjaunias et al. [2]	1975	Selective angiography and microradiographs of injected specimens	Deep maxillary supply to orbit via MMA, anterior deep temporal, and infraorbital arteries	Narrative anatomical synthesis
Müller [10]	1978	Macroscopic study with microscopic verification	Anterior falcate artery connections with MMA and collateral relevance to orbit	Narrative only
Ducasse et al. [7]	1984	Latex injection and orbital dissection	Lacrimal gland arterial supply from OA and MMA	Qualitative source derivation
Ducasse et al. [8]	1985	Anatomical dissection	ECA participation in orbital vascularization, especially meningo-lacrimal and sphenoidal pathways	Exploratory/ supplementary
Diamond [4]	1991	Dissection plus comparative osteology	Meningolacrimal versus sphenoidal artery and meningo-orbital routes	Exploratory/narrative
Shimada et al. [6]	1995	Cadaveric dissection with vascular injection in subseries	Orbital branch of MMA via superior orbital fissure or meningo-orbital foramen	Narrative/subgroup only
Konishi and Kikuchi [9]	1996	Macroscopic anatomical dissection and classification	*R. anastomoticus* cum a. lacrimali/recurrent meningeal-lacrimal route	Exploratory quantitative/recurrent meningeal route
Liu and Rhoton [5]	2001	Microsurgical dissection	OA arising from MMA; recurrent lacrimal-MMA connections	Narrative anatomical synthesis
Perrini et al. [3]	2007	Microsurgical dissection	Course of OA and dangerous anastomoses with MMA	Exploratory/supplementary family-specific synthesis
Bracco et al. [18]	2016	Angiographic study across 443 procedures	Transorbital ICA-ECA anastomoses including lacrimal-MMA and OA-MMA	Exploratory/supplementary
Ondas et al. [16]	2020	3D time-of-flight magnetic resonance angiography	Anomalous origin of OA, including OA from MMA	Narrative/descriptive
Aktaş et al. [17]	2020	Superselective ophthalmic artery angiography	Direct OA-MMA anastomoses, recurrent meningeal branch, anterior falx artery	Exploratory/narrative
Fantoni et al. [11]	2020	Selective carotid angiography	OA origin of MMA in cSDH	Primary plus sensitivity analysis
Shotar et al. [12]	2021	DSA with CT/CTA review when available	Angiographic relationship between MMA and OA, including meningo-lacrimal and sphenoidal patterns	Primary, exploratory, or narrative depending on anatomical family
Sari et al. [13]	2023	3D rotational angiography, MIP and CBCT	MMA-OA relationship and variant origins	Primary or narrative depending on anatomical family
Pilawska et al. [14]	2024	Digital subtraction angiography	Variants of MMA, including OA origin of MMA	Primary quantitative
Hubbard et al. [15]	2025	Preprocedural CT plus angiographic review	Ophthalmic contribution to MMA system and feasibility of embolization	Qualitative/narrative
Senol et al. [19]	2025	Microcatheterization, contrast injection, angiography and DynaCT	Meningo-lacrimal anastomosis/orbital branches of MMA	Exploratory/supplementary family-specific synthesis

Note. Detailed extraction variables and source derivation are provided in the Supplementary Material.

**Table 2:** Route-specific anatomical definitions used for classification and synthesis.

Anatomical Family	Operational Definition Used in This Review	Synthesis Interpretation
MMA from OA	Middle meningeal artery arising from the ophthalmic arterial system rather than from the maxillary artery.	Primary quantitative analysis when numerator, denominator, and unit of analysis were compatible.
OA from MMA	Ophthalmic artery arising completely or partially from the middle meningeal artery or external carotid route.	Narrative/descriptive because independent comparable denominators were limited.
Direct OA-MMA anastomosis	Direct collateral channel between the ophthalmic artery and middle meningeal artery without complete substitution of arterial origin.	Exploratory only because definitions and populations were heterogeneous.
Meningolacrimal/lacrimal-MMA route	Communication between lacrimal/orbital branches and the middle meningeal artery, including meningolacrimal artery patterns.	Exploratory/supplementary because cadaveric and angiographic detectability differed substantially.
Recurrent meningeal route	Recurrent meningeal branch or orbitomeningeal vessel coursing from the lacrimal/ophthalmic territory toward the dura or MMA territory.	Exploratory only because methods and denominators were heterogeneous.
Sphenoidal route	Sphenoidal branch or foraminal orbital route linking the MMA with lacrimal, lateral muscular, or ophthalmic territories.	Narrative/descriptive because few comparable studies were available.
Anterior falx route	Falx-related collateral pathway involving anterior ethmoidal/ophthalmic contributions to anterior dural or falcine branches.	Narrative only; no independent R-ready family was available.

Note. These operational definitions were used to preserve route-specific anatomical meaning during extraction and poolability assessment. They are not intended to replace detailed embryological or surgical classifications.

**Table 3:** Family-specific poolability decisions.

Family	Evidence Base	Decision and Use
MMA from OA	5 rows; 4 studies; 16/537	Primary quantitative; main analysis
MLA/lacrimal-MMA route	6 rows; 6 studies; 89/359	Exploratory quantitative; supplementary
Direct OA-MMA	3 rows; 3 studies; 37/372	Exploratory only; if reported
Recurrent meningeal route	3 rows; 3 studies; 258/440	Exploratory only; if reported
OA from MMA	2 rows; 2 studies; 57/16,111	Narrative/descriptive; no meta-analysis
Sphenoidal route	2 rows; 2 studies; 42/210	Narrative/descriptive; no meta-analysis
Anterior falx route	No independent quantitative rows	Narrative only; no quantitative data

Note. Unpooled event and denominator sums are descriptive only and should not be interpreted as pooled prevalence estimates.

**Table 4:** Quantitative synthesis, sensitivity analysis, and descriptive subgroup results.

Analysis	Role and Evidence Base	Estimate and Prediction Interval	Heterogeneity	Use
MMA from OA	Primary; 5 rows/4 studies; 16/537	0.03 (95% CI 0.01 to 0.13); PI 0.00 to 0.49	I^2^ = 75.9%; tau^2^ = 1.2534; *p* = 0.001	Main figure
MMA from OA, excluding cSDH	Sensitivity; 4 rows/4 studies; 8/479	0.02 (95% CI 0.01 to 0.06); PI 0.01 to 0.07	I^2^ = 7.7%; tau^2^ = 0.0487; *p* = 0.379	Sensitivity
MLA/lacrimal-MMA	Exploratory; 6 rows/6 studies; 89/359	0.45 (95% CI 0.09 to 0.86); PI 0.01 to 0.99	I^2^ = 95.9%; tau^2^ = 3.1949; *p* < 0.001	Supplementary
MLA cadaveric/ anatomic	Exploratory subgroup; 4 rows/4 studies; 51/113	0.66 (95% CI 0.07 to 0.98); PI not estimated separately	I^2^ = 89.9%; tau^2^ = 3.4541; *p* < 0.001	Figure 3; descriptive
MLA imaging/ angiographic	Exploratory subgroup; 2 rows/2 studies; 38/246	0.16 (95% CI 0.00 to 0.94); PI not estimated separately	I^2^ = 74.5%; tau^2^ = 0.1866; *p* = 0.048	Figure 3; descriptive

Note. Pooled estimates were calculated only for family-specific and sensitivity analyses considered methodologically defensible after row-level poolability audit.

**Table 5:** Summary of JBI-informed methodological-quality appraisal.

JBI-Informed Judgement Category	Number of Studies	Interpretation
Low/some concerns	6	Generally clear denominator and method, but with anatomical or clinical selection caveats.
Some concerns	7	Useful anatomical evidence with limited sample size, conditional denominators, or partial reporting constraints.
High/some concerns or high	6	Selected clinical/case-based/historical evidence mainly retained for narrative or exploratory interpretation.

Note. The detailed study-level and item-level appraisal is provided in Supplementary Table S3. The categories low/some concerns, some concerns, and high/some concerns are interpretive synthesis categories derived from the JBI-informed appraisal and are not formal RoB 2 judgments.

## Data Availability

All data supporting the qualitative and quantitative syntheses are provided in the manuscript and Supplementary Material, including the final independent-row quantitative matrix and the dropped/merged row audit. The final extraction matrix and R-ready matrix are provided in the Supplementary Material. The analysis code is available from the corresponding author upon request.

## Supplementary Materials

The following supporting information can be downloaded at: https://www.mdpi.com/article/10.3390/neurolint18070128/s1. Supplementary Table S1: Search strategies and source yields; Supplementary Table S2: Full-text excluded studies; Supplementary Table S2a: Duplicate reports identified at full-text stage; Supplementary Table S3: Detailed JBI item-level appraisal; Supplementary Table S4: Final independent R-ready matrix; Supplementary Table S5: Dropped or merged rows during final poolability audit; Supplementary Table S6: Quantitative synthesis, sensitivity, and subgroup results; Supplementary Table S7: PRISMA 2020 checklist; Supplementary Figure S1: Meningolacrimal/lacrimal-MMA route across eligible series [3,4,8,12,18,19]. Exploratory family-specific random-effects meta-analysis using logit-transformed proportions, restricted maximum likelihood estimation, and Hartung-Knapp confidence intervals. This estimate is supplementary and should not be interpreted as a stable general-population prevalence; Supplementary Figure S2: Sensitivity analysis for MMA arising from OA excluding the clinically selected cSDH subgroup [11,12,13,14]. Random-effects meta-analysis using logit-transformed proportions, restricted maximum likelihood estimation, and Hartung-Knapp confidence intervals.

## Abbreviations

**Abbreviation**	**Definition**
cSDH	chronic subdural hematoma
DSA	digital subtraction angiography
ECA	external carotid artery
ICA	internal carotid artery
JBI	Joanna Briggs Institute
MMA	middle meningeal artery
OA	ophthalmic artery
PRISMA	Preferred Reporting Items for Systematic Reviews and Meta-Analyses
REML	restricted maximum likelihood
