# Supplementary material for: Route-Specific Meningo-Ophthalmic and Orbitomeningeal Communications Relevant to Middle Meningeal Artery Embolization: A Systematic Review and Meta-Analysis
Source: Neurol Int. 2026 Jul 6;18(7):128. doi: 10.3390/neurolint18070128 (PMC13414621; doi:10.3390/neurolint18070128)
Supplement: Supplementary file 1 [file neurolint-18-00128-s001.zip › neurolint-4373130-supplementary.pdf]

Supplementary Material

Route-specific meningo-ophthalmic and orbitomeningeal communications relevant to middle meningeal artery embolization: a systematic review and meta-analysis

Supplementary reference key for included studies

Ref.	Study	Role in synthesis
[1]	McLennan et al. (1974)	Qualitative anatomical synthesis
[2]	Lasjaunias et al. (1975)	Qualitative anatomical synthesis
[3]	Perrini et al. (2007)	MLA/recurrent route quantitative/exploratory
[4]	Diamond (1991)	MLA/lacrimal-MMA quantitative/exploratory
[5]	Liu & Rhoton (2001)	Qualitative anatomical synthesis
[6]	Shimada et al. (1995)	Narrative/conditional anatomical evidence
[7]	Ducasse et al. (1984)	Qualitative/anatomical evidence
[8]	Ducasse et al. (1985)	MLA/lacrimal-MMA quantitative/exploratory
[9]	Konishi & Kikuchi (1996)	Recurrent meningeal exploratory evidence
[10]	Müller (1978)	Anterior falx route/narrative
[11]	Fantoni et al. (2020)	MMA from OA primary and sensitivity evidence
[12]	Shotar et al. (2021/2022)	MMA from OA, MLA, direct and sphenoidal evidence
[13]	Sari et al. (2023)	MMA from OA and OA from MMA evidence
[14]	Pilawska et al. (2024)	MMA from OA primary evidence
[15]	Hubbard et al. (2025)	Qualitative/narrative procedural evidence
[16]	Ondas et al. (2019/2020)	OA from MMA descriptive evidence
[17]	Aktaş et al. (2020)	Direct/recurrent route exploratory evidence
[18]	Bracco et al. (2016)	MLA/direct OA-MMA exploratory evidence
[19]	Senol et al. (2025)	MLA/lacrimal-MMA quantitative/exploratory evidence

Supplementary Table S1. Search strategies and source yields

Note. The strategies below are reproduced from the validated search strategy document. No publication-year limits were applied. Exact search dates are reported for each database. No automated language, human-participant, or document-type filters were documented/applied in the recorded strategies available for audit.

Database / platform	Search strategy	Limits / notes	Records retrieved	Comments
PubMed (MEDLINE)	(("middle meningeal artery"[Title/Abstract] OR "middle meningeal arteries"[Title/Abstract] OR "meningeal artery"[Title/Abstract] OR "meningeal arteries"[Title/Abstract] OR "meningolacrimal	Search date: 9 May 2026. No date restriction. Human and language filters were not prespecified in the working notes available. No automated language, human-participant,	140	Built to capture direct MMA–OA/lacrimal relationships and collateral/anomalous origin patterns.

Database / platform	Search strategy	Limits / notes	Records retrieved	Comments
	artery"[Title/Abstract] OR "meningo-lacrimal artery"[Title/Abstract] OR "meningolacrimal branch"[Title/Abstract] OR "meningoorbital artery"[Title/Abstract] OR "meningo-orbital artery"[Title/Abstract] OR "sphenoidal artery"[Title/Abstract] OR "recurrent meningeal artery"[Title/Abstract] OR "orbital branch of the middle meningeal artery"[Title/Abstract] OR "orbital branch"[Title/Abstract] OR "anterior falcate artery"[Title/Abstract] OR "falx artery"[Title/Abstract]) AND ("ophthalmic artery"[Title/Abstract] OR "ophthalmic arteries"[Title/Abstract] OR "lacrimal artery"[Title/Abstract] OR "lacrimal arteries"[Title/Abstract] OR orbit*[Title/Abstract] OR orbital[Title/Abstract] OR orbita*[Title/Abstract] OR ophthalmic[Title/Abstract] OR ethmoidal[Title/Abstract])) OR (("middle meningeal artery"[MeSH Terms] OR "ophthalmic artery"[MeSH Terms]) AND (anastomo*[Title/Abstract] OR origin*[Title/Abstract] OR variant*[Title/Abstract] OR collateral*[Title/Abstract] OR branch*[Title/Abstract] OR communication*[Title/Abstract] OR supply[Title/Abstract] OR vascularization[Title/Abstract] OR vascularisation[Title/Abstract]))	or document-type filters were documented/applied in the recorded strategy.		
Scopus	TITLE-ABS-KEY(("middle meningeal artery" OR "middle meningeal arteries" OR "meningolacrimal artery" OR "meningo-lacrimal artery" OR "meningoorbital artery" OR "meningo-orbital artery" OR "sphenoidal artery" OR "recurrent meningeal artery" OR "orbital branch of the middle meningeal artery" OR "anterior falcate artery" OR "falx artery") AND ("ophthalmic artery" OR "ophthalmic arteries" OR "lacrimal artery" OR "lacrimal arteries" OR orbit* OR orbital OR orbita* OR ophthalmic OR ethmoidal) AND (anastomo* OR origin* OR variant* OR collateral* OR branch* OR communication* OR supply OR vascularization OR vascularisation))	Search date: 9 May 2026. No year restriction documented in current files. Search run in title/abstract/keywords field. No automated language, human-participant, or document-type filters were documented/applied in the recorded strategy.	181	Adapted for broad recall in multidisciplinary indexing.
Web of Science Core Collection	TS=((("middle meningeal artery" OR "middle meningeal arteries" OR "meningolacrimal artery" OR "meningo-lacrimal artery" OR "meningoorbital artery" OR "meningo-orbital artery" OR "sphenoidal artery" OR "recurrent meningeal artery" OR "orbital branch of the middle meningeal artery" OR "anterior falcate artery" OR "falx artery") NEAR/3 ("ophthalmic artery" OR "ophthalmic arteries" OR "lacrimal artery" OR "lacrimal arteries" OR orbit* OR orbital OR orbita* OR ophthalmic	Search date: 9 May 2026. Core Collection search. No date restriction documented in current files. No automated language, human-participant, or document-type filters were documented/applied in the recorded strategy.	120	Uses proximity to improve specificity while retaining broad variant terminology.

Database / platform	Search strategy	Limits / notes	Records retrieved	Comments
	OR ethmoidal)) OR (("middle meningeal artery" OR "ophthalmic artery") AND (anastomo* OR origin* OR variant* OR collateral* OR branch* OR communication* OR supply OR vascularization OR vascularisation)))			
CINAHL (EBSCOhost)	((TI ("middle meningeal artery" OR "middle meningeal arteries" OR "meningolacrimal artery" OR "meningo-lacrimal artery" OR "meningoorbital artery" OR "meningo-orbital artery" OR "sphenoidal artery" OR "recurrent meningeal artery" OR "orbital branch of the middle meningeal artery" OR "anterior falcate artery" OR "falx artery")<br>OR AB ("middle meningeal artery" OR "middle meningeal arteries" OR "meningolacrimal artery" OR "meningo-lacrimal artery" OR "meningoorbital artery" OR "meningo-orbital artery" OR "sphenoidal artery" OR "recurrent meningeal artery" OR "orbital branch of the middle meningeal artery" OR "anterior falcate artery" OR "falx artery"))<br>AND<br>(TI ("ophthalmic artery" OR "ophthalmic arteries" OR "lacrimal artery" OR "lacrimal arteries" OR orbit* OR orbital OR orbita* OR ophthalmic OR ethmoidal)<br>OR AB ("ophthalmic artery" OR "ophthalmic arteries" OR "lacrimal artery" OR "lacrimal arteries" OR orbit* OR orbital OR orbita* OR ophthalmic OR ethmoidal))<br>AND<br>(TI (anastomo* OR origin* OR variant* OR collateral* OR branch* OR communication* OR supply OR vascularization OR vascularisation)<br>OR AB (anastomo* OR origin* OR variant* OR collateral* OR branch* OR communication* OR supply OR vascularization OR vascularisation)))	Search date: 9 May 2026. No date restriction documented in current files. No automated language, human-participant, or document-type filters were documented/applied in the recorded strategy.	114	Title/abstract strategy adapted to EBSCO syntax.
LILACS (BVS)	(tw:("middle meningeal artery" OR "middle meningeal arteries" OR "meningolacrimal artery" OR "meningo-lacrimal artery" OR "meningoorbital artery" OR "meningo-orbital artery" OR "sphenoidal artery" OR "recurrent meningeal artery" OR "orbital branch of the middle meningeal artery" OR "anterior falcate artery" OR "falx artery")<br>AND<br>tw:("ophthalmic artery" OR "ophthalmic arteries" OR "lacrimal artery" OR "lacrimal arteries" OR orbit* OR orbital OR orbita* OR ophthalmic OR ethmoidal)<br>AND<br>tw:(anastomo* OR origin* OR variant* OR collateral* OR branch* OR communication* OR supply OR	Search date: 9 May 2026. No date restriction documented in current files. No automated language, human-participant, or document-type filters were documented/applied in the recorded strategy.	3	Conservative strategy given low expected yield; can be expanded with Portuguese/Spanish synonyms if rerun is required.

Database / platform	Search strategy	Limits / notes	Records retrieved	Comments
	vascularization OR vascularisation)<br>)			

Supplementary Table S2. Full-text excluded studies

Note. Duplicate reports were handled separately during study consolidation and are not included as exclusions in this table.

No.	Study title	Standardized exclusion reason	Brief argument for exclusion
3	Ophthalmic artery arising from the external carotid artery system: the middle meningeal artery in South African patients	Wrong study design / case report only	Two-case report (one adult, one child) without a valid source population or prevalence denominator.
4	Anatomic and Embryologic Analysis of the Dural Branches of the Ophthalmic Artery	Wrong study design / review article	Review article; does not present an eligible original cohort for prevalence/anatomy synthesis.
5	Accessory middle meningeal artery or anastomosis between the ophthalmic and the middle meningeal arteries? On the correct way to make a proper identification	Wrong study design / letter-commentary without original dataset	Letter to the editor/commentary; conceptually useful but no original eligible dataset.
7	Middle meningeal artery embolization contraindication when it originates from the ophthalmic artery	Wrong study design / image report or single-case illustration	Brief illustrative image/case report without denominator or systematic anatomical series.
9	Endoscopic cadaveric analysis of the origin of the ophthalmic artery	Wrong anatomical focus	Focuses on OA origin from ICA and endonasal relationships, not on MMA–OA/anastomotic outcomes of interest.
11	Sphenoidal artery: review of the literature and analysis of a dissected arterially injected fetal orbit	Wrong study design / review with narrative analysis, not an eligible original prevalence series	Predominantly a review with only single fetal specimen analysis; not a comparable original adult/cadaveric/radiologic prevalence series.
14	Embryology and anatomical variations of the ophthalmic artery	Wrong study design / review article	Review article without original cohort data.
16	Anatomic and Angiographic Analyses of Ophthalmic Artery Collaterals in Moyamoya Disease	Wrong population / wrong clinical-anatomical focus	Study of OA collaterals in Moyamoya; one incidental lacrimal–MMA case only, not designed around the review question.
18	Anatomy of the Ophthalmic Artery: Embryological Consideration	Wrong study design / review article	Review article used for background/embryology, not eligible as primary study.
19	Different Ophthalmic Artery Origins: Embryology and Clinical Significance	Wrong study design / review article	Literature-based retrospective review rather than an original cohort.
20	Impact of anomalous origin of the ophthalmic artery from the middle meningeal artery on selection of surgical approach to skull base meningioma	Wrong study design / case series without eligible prevalence framework / surgical illustrative series	Three-case surgical illustrative series without population denominator.
22	Orbital vascular anatomy	Wrong study design / narrative review	Narrative/synthesis paper, valuable for discussion but not an original eligible study for the main evidence set.
32	Selective External Carotid Angiography and its Clinical Applications	Wrong study design / imaging-clinical series not eligible for anatomical synthesis	Clinical angiography applications series; includes relevant examples but not a systematic anatomical prevalence study.

Supplementary Table S2a. Duplicate reports identified at full-text stage

Duplicate record	Master record	Study title	Decision/handling
26	25	Middle Meningeal Origin of the Ophthalmic Artery	Duplicate report; handled separately from exclusions
31b	31	Homologies of the meningeal-orbital arteries of humans: a reappraisal	Duplicate report; handled separately from exclusions
38	37	Anterior falcate artery in the adult / Die Arteria falcea anterior des Erwachsenen	Duplicate report; handled separately from exclusions

Supplementary Table S3. Detailed JBI item-level appraisal

Note. Y = yes/low concern; U = unclear; N = no/high concern; N/U = no or unclear. The appraisal was used to guide synthesis role, not as an automatic exclusion rule. The overall categories low/some concerns, some concerns, and high/some concerns are interpretive synthesis categories derived from this JBI-informed prevalence appraisal and are not formal RoB 2 judgments.

Ref.	Study	D1 Sample frame	D2 Sampling	D3 Sample size	D4 Subjects/setting	D5 Coverage	D6 Valid identification	D7 Standard measurement	D8 Analysis	D9 Complete data	Overall
[19]	Senol et al. (2025)	Y	U	U	Y	U	Y	Y	Y	U	Some concerns
[13]	Sari et al. (2023)	Y	Y	Y	Y	Y	Y	Y	Y	Y	Low/some concerns
[15]	Hubbard et al. (2025)	Y	U	U	Y	U	Y	Y	Y	U	Some concerns
[14]	Pilawska et al. (2024)	Y	Y	Y	Y	Y	Y	Y	Y	Y	Low/some concerns
[12]	Shotar et al. (2021)	Y	Y	Y	Y	Y	Y	Y	Y	Y	Low/some concerns
[11]	Fantoni et al. (2020)	Y	U	U	Y	U	Y	Y	Y	U	Some concerns
[17]	Aktaş et al. (2020)	U	N/U	N/U	Y/U	U	Y/U	U	N/U	U	High/some concerns
[16]	Ondas et al. (2019)	Y	Y	Y	Y	Y	Y	Y	Y	Y	Low/some concerns
[18]	Bracco et al. (2016)	U	N/U	N/U	Y/U	U	Y/U	U	N/U	U	High/some concerns
[3]	Perrini et al. (2007)	Y	U	U	Y	U	Y	Y	Y	U	Some concerns
[5]	Liu & Rhoton (2001)	U	N/U	N/U	Y/U	U	Y/U	U	N/U	U	High/some concerns
[6]	Shimada et al. (1995)	Y	U	U	Y	U	Y	Y	Y	U	Some concerns

Ref.	Study	D1 Sample frame	D2 Sampling	D3 Sample size	D4 Subjects/setting	D5 Coverage	D6 Valid identification	D7 Standard measurement	D8 Analysis	D9 Complete data	Overall
[4]	Diamond (1991)	U	N/U	N/U	Y/U	U	Y/U	U	N/U	U	High/some concerns
[8]	Ducasse et al. (1985)	Y	Y	Y	Y	Y	Y	Y	Y	Y	Low/some concerns
[7]	Ducasse et al. (1984)	Y	U	U	Y	U	Y	Y	Y	U	Some concerns
[10]	Müller (1978)	U	N/U	N/U	Y/U	U	Y/U	U	N/U	U	High/some concerns
[2]	Lasjaunias et al. (1975)	Y	U	U	Y	U	Y	Y	Y	U	Some concerns
[1]	McLennan et al. (1974)	U	N/U	N/U	Y/U	U	Y/U	U	N/U	U	High/some concerns
[9]	Konishi & Kikuchi (1996)	Y	Y	Y	Y	Y	Y	Y	Y	Y	Low/some concerns

Supplementary Table S4. Final independent R-ready matrix

Note. Rows are independent after final poolability audit. The MLA/lacrimal-MMA family is labelled as exploratory/supplementary in line with the main manuscript interpretation.

Ref.	Study	Year	Event	Family	Analysis tier	Events	N	Unit	Evidence source	Modality group	Independence rationale
[11]	Fantoni et al. (2020)	2020	Ophthalmic origin of MMA in cSDH group	MMA_from_OA	Primary quantitative synthesis	8	58	arteries	Imaging/angiographic	Selective carotid angiography	Independent study subgroup from Fantoni et al.; cSDH and control groups are distinct denominators.
[11]	Fantoni et al. (2020)	2020	Ophthalmic origin of MMA in control group	MMA_from_OA	Primary quantitative synthesis	1	131	arteries	Imaging/angiographic	Selective carotid angiography	Independent study subgroup from Fantoni et al.; cSDH and control groups are distinct denominators.
[12]	Shotar et al. (2021)	2021	Complete ophthalmic origin of MMA	MMA_from_OA	Primary quantitative synthesis	1	140	MMAs	Imaging/angiographic	DSA ± CT/CTA	Independent denominator/event for MMA arising from ophthalmic artery.
[13]	Sari et al. (2023)	2023	MMA arising from OA	MMA_from_OA	Primary quantitative synthesis	2	87	hemispheres	Imaging/angiographic	3D rotational angiography	Independent denominator/event for MMA arising from ophthalmic artery.
[14]	Pilawska et al. (2024)	2024	MMA arising from OA	MMA_from_OA	Primary quantitative synthesis	4	121	MMAs	Imaging/angiographic	DSA	Independent denominator/event for MMA arising from ophthalmic artery.

Ref.	Study	Year	Event	Family	Analysis tier	Events	N	Unit	Evidence source	Modality group	Independence rationale
[8]	Ducasse et al. (1985)	1985	Any meningolacrimal artery pattern (combined extracted categories)	MLA_lacrimal_MMA	Exploratory/supplementary quantitative synthesis	20	70	dissections	Cadaveric/anatomic	Anatomical dissection	Original two rows shared the same denominator and represented non-independent subcategories; combined to one study-level event.
[4]	Diamond (1991)	1991	Any meningolacrimal artery pattern (combined extracted categories)	MLA_lacrimal_MMA	Exploratory/supplementary quantitative synthesis	12	13	orbital dissections	Cadaveric/anatomic	Dissection + osteology	Original two rows shared the same denominator and represented non-independent subcategories; combined to one study-level event.
[3]	Perrini et al. (2007)	2007	Meningolacrimal artery pattern	MLA_lacrimal_MMA	Exploratory/supplementary quantitative synthesis	4	14	orbits	Cadaveric/anatomic	Microsurgical dissection	Independent denominator/event for meningolacrimal/lacrimal-MMA route.
[18]	Bracco et al. (2015)	2016	Lacrimal artery–MMA anastomosis	MLA_lacrimal_MMA	Exploratory/supplementary quantitative synthesis	22	106	orbits	Imaging/angiographic	Orbital angiography	Independent denominator/event for meningolacrimal/lacrimal-MMA route.
[12]	Shotar et al. (2021)	2021	Meningolacrimal artery	MLA_lacrimal_MMA	Exploratory/supplementary quantitative synthesis	16	140	MMAs	Imaging/angiographic	DSA ± CT/CTA	Independent denominator/event for meningolacrimal/lacrimal-MMA route.
[19]	Senol et al. (2025)	2025	Meningolacrimal anastomosis visualized	MLA_lacrimal_MMA	Exploratory/supplementary quantitative synthesis	15	16	MMA specimens	Cadaveric/anatomic	Cadaveric angiography	Independent denominator/event for meningolacrimal/lacrimal-MMA route.
[18]	Bracco et al. (2015)	2016	OA–MMA anastomosis	Direct_OA_MMA	Exploratory quantitative synthesis	15	106	orbits	Imaging/angiographic	Orbital angiography	Independent denominator/event for direct OA-MMA anastomotic communication; heterogeneous adult/pediatric and angiographic settings.
[17]	Aktas et al. (2020)	2020	Anastomotic branch with MMA visible	Direct_OA_MMA	Exploratory quantitative synthesis	15	126	angiographies	Imaging/angiographic	Superselective OA angiography	Independent denominator/event for direct OA-MMA anastomotic communication; heterogeneous adult/pediatric and angiographic settings.
[12]	Shotar et al. (2021)	2021	Global MMA–OA trunk anastomosis	Direct_OA_MMA	Exploratory quantitative synthesis	7	140	MMAs	Imaging/angiographic	DSA ± CT/CTA	Independent denominator/event for direct OA-MMA

Ref.	Study	Year	Event	Family	Analysis tier	Events	N	Unit	Evidence source	Modality group	Independence rationale
											anastomotic communication; heterogeneous adult/pediatric and angiographic settings.
[9]	Konishi & Kikuchi (1996)	1996	R. anastomoticus cum a. lacrimali present	Recurrent_meningeal	Exploratory quantitative synthesis	200	300	orbits	Cadaveric/anatomic	Anatomical dissection	Full-text abstract/table report seven types among 300 orbits; non-absent types sum to 200/300.
[3]	Perrini et al. (2007)	2007	Recurrent meningeal anastomosis with OA system	Recurrent_meningeal	Exploratory quantitative synthesis	11	14	orbits	Cadaveric/anatomic	Microsurgical dissection	Independent denominator/event for recurrent meningeal route; heterogeneous methods and populations.
[17]	Aktas et al. (2020)	2020	Recurrent meningeal branch visible	Recurrent_meningeal	Exploratory quantitative synthesis	47	126	angiographies	Imaging/angiographic	Superselective OA angiography	Independent denominator/event for recurrent meningeal route; heterogeneous methods and populations.
[16]	Ondas et al. (2019)	2019	Any OA arising from MMA (at least one side)	OA_from_MMA	Narrative/descriptive only	56	16024	patients	Imaging/angiographic	TOF-MRA	Independent patient-level global event; laterality rows are nested and excluded.
[13]	Sari et al. (2023)	2023	OA completely arising from MMA	OA_from_MMA	Narrative/descriptive only	1	87	hemispheres	Imaging/angiographic	3D rotational angiography	Independent study-level event; retained for descriptive tabulation.
[8]	Ducasse et al. (1985)	1985	Sphenoidal artery anastomosing MMA to lacrimal/lateral muscular branch	Sphenoidal_route	Narrative/descriptive only	38	70	dissections	Cadaveric/anatomic	Anatomical dissection	Independent route-specific event but insufficient number of comparable studies for formal prevalence synthesis.
[12]	Shotar et al. (2021)	2021	Sphenoidal branch with anastomosis to OA trunk	Sphenoidal_route	Narrative/descriptive only	4	140	MMAs	Imaging/angiographic	DSA ± CT/CTA	Independent route-specific event but insufficient number of comparable studies for formal prevalence synthesis.

Supplementary Table S5. Dropped or merged rows during final poolability audit

Note. Rows were dropped or merged when they were duplicative, nested, conditional, or otherwise non-independent for the intended family-specific quantitative analyses.

Ref.	original_event_id	study_label	year	original_event_label	meta_family_code	events	total_n	final_decision	reason
[13]	4	Sari et al. (2023)	2023	MMA arising from OA + maxillary artery	MMA_from_OA	2	87	Excluded from R input	Duplicative/non-independent with Sari 2023 MMA_from_OA row sharing same denominator and event count.
[12]	6	Shotar et al. (2021)	2021	Any angiographic relation between MMA and OA	Direct_OA_MMA	26	140	Excluded from R input	Broad composite 'any relation' overlaps with route-specific Shotar rows; keep global OA-MMA row only for Direct_OA_MMA.
[12]	10	Shotar et al. (2021)	2021	Patent meningolacrimal-to-OA trunk anastomosis	MLA_lacrimal_MMA	3	16	Excluded from R input	Conditional denominator among MLA-positive MMAs; not comparable with global prevalence rows.
[16]	17	Ondas et al. (2019)	2019	Right OA arising from MMA	OA_from_MMA	28	16024	Excluded from R input	Laterality row nested within Ondas 2019 global any-side row.
[16]	18	Ondas et al. (2019)	2019	Left OA arising from MMA	OA_from_MMA	16	16024	Excluded from R input	Laterality row nested within Ondas 2019 global any-side row.
[16]	19	Ondas et al. (2019)	2019	Bilateral OA arising from MMA	OA_from_MMA	12	16024	Excluded from R input	Bilateral row nested within Ondas 2019 global any-side row.
[6]	24	Shimada et al. (1995)	1995	Type 4 among MOF cases	Direct_OA_MMA	4	116	Narrative/conditional only	Conditional MOF-positive denominator; not comparable with general prevalence denominators.
[6]	25	Shimada et al. (1995)	1995	Anastomosis among SOF cases	Direct_OA_MMA	12	20	Narrative/conditional only	Conditional SOF-positive denominator; not comparable with general prevalence denominators.
[4]	26	Diamond (1991)	1991	Meningolacrimal artery supplying	MLA_lacrimal_MMA	7	13	Merged	Merged with original event 27 to

Ref.	original_event_id	study_label	year	original_event_label	meta_family_code	events	total_n	final_decision	reason
				lacrimal territory as sole branch					create independent combined Diamond row 31.
[4]	27	Diamond (1991)	1991	Dual persistence of sphenoidal + meningolacrimal artery	MLA_lacrimal_MMA	5	13	Merged	Merged with original event 26 to create independent combined Diamond row 31.
[8]	28	Ducasse et al. (1985)	1985	Meningolacrimal artery as sole lacrimal artery	MLA_lacrimal_MMA	12	70	Merged	Merged with original event 29 to create independent combined Ducasse row 32.
[8]	29	Ducasse et al. (1985)	1985	Meningolacrimal artery with another classical lacrimal artery	MLA_lacrimal_MMA	8	70	Merged	Merged with original event 28 to create independent combined Ducasse row 32.

Supplementary Table S6. Quantitative synthesis, sensitivity, and subgroup results

Note. Study references identify the included studies contributing to each quantitative analysis. Subgroup estimates are descriptive and should not be interpreted as confirmatory tests of subgroup differences. Analyses were conducted using the software and package versions reported in the main manuscript.

Analysis	Role	Study references	Rows	Studies/subgroups	Events/N	Pooled proportion	95% CI	Prediction interval	I^2	tau²	Heterogeneity p	Interpretation
MMA arising from ophthalmic artery	Primary	[11-14]	5	4	16/537	0.03	0.01 to 0.13	0.00 to 0.49	75.9%	1.2534	0.001	Main quantitative figure
MLA/lacrimal-MMA route	Exploratory/supplementary	[3,4,8,12,18,19]	6	6	89/359	0.45	0.09 to 0.86	0.01 to 0.99	95.9%	3.1949	<0.001	Supplementary/exploratory
MMA arising from ophthalmic artery, excluding Fantoni cSDH	Sensitivity	[11-14]	4	4	8/479	0.02	0.01 to 0.06	0.01 to 0.07	7.7%	0.0487	0.379	Sensitivity analysis
MLA/lacrimal-MMA route, cadaveric/anatomic evidence	Exploratory subgroup	[3,4,8,19]	4	4	51/113	0.66	0.07 to 0.98	Not estimated separately	89.9%	3.4541	<0.001	Descriptive subgroup
MLA/lacrimal-MMA route, imaging/angiographic evidence	Exploratory subgroup	[12,18]	2	2	38/246	0.16	0.00 to 0.94	Not estimated separately	74.5%	0.1866	0.048	Descriptive subgroup

Supplementary Figures

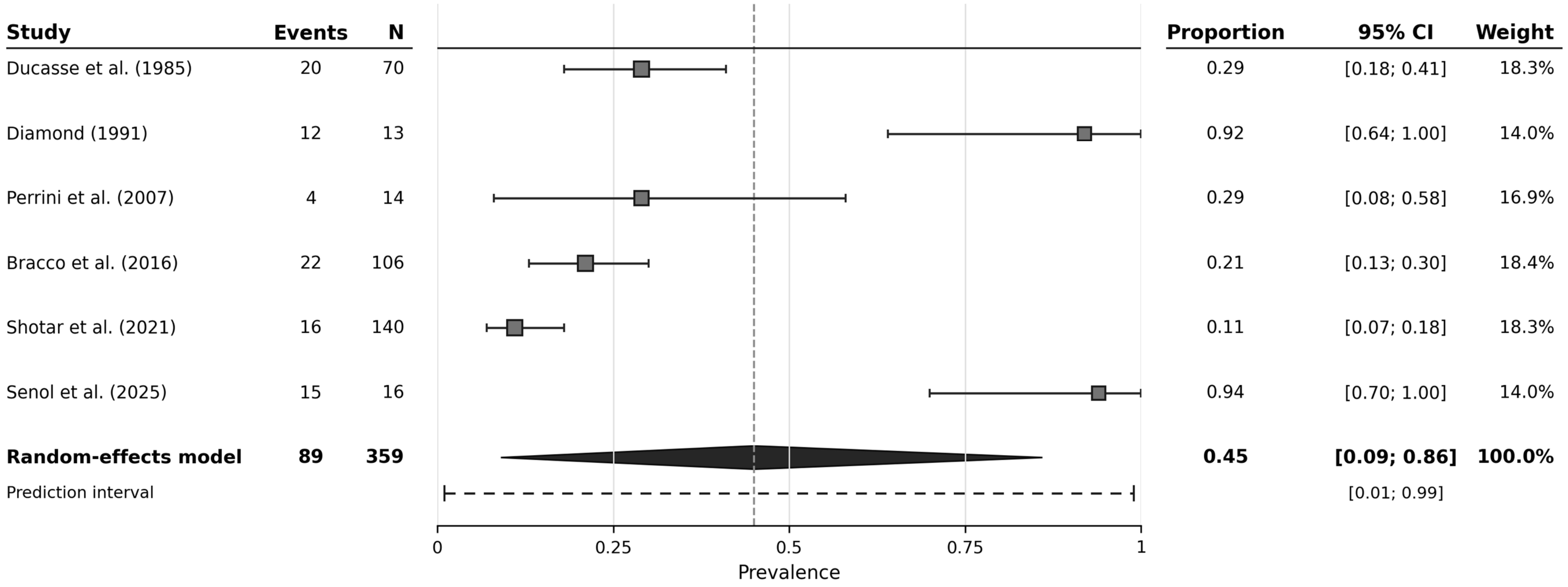

Figure S1. Meningolacrimal/lacrimal-MMA route across eligible series [3,4,8,12,18,19]. Exploratory family-specific random-effects meta-analysis using logit-transformed proportions, restricted maximum likelihood estimation, and Hartung-Knapp confidence intervals. This estimate is supplementary and should not be interpreted as a stable general-population prevalence.

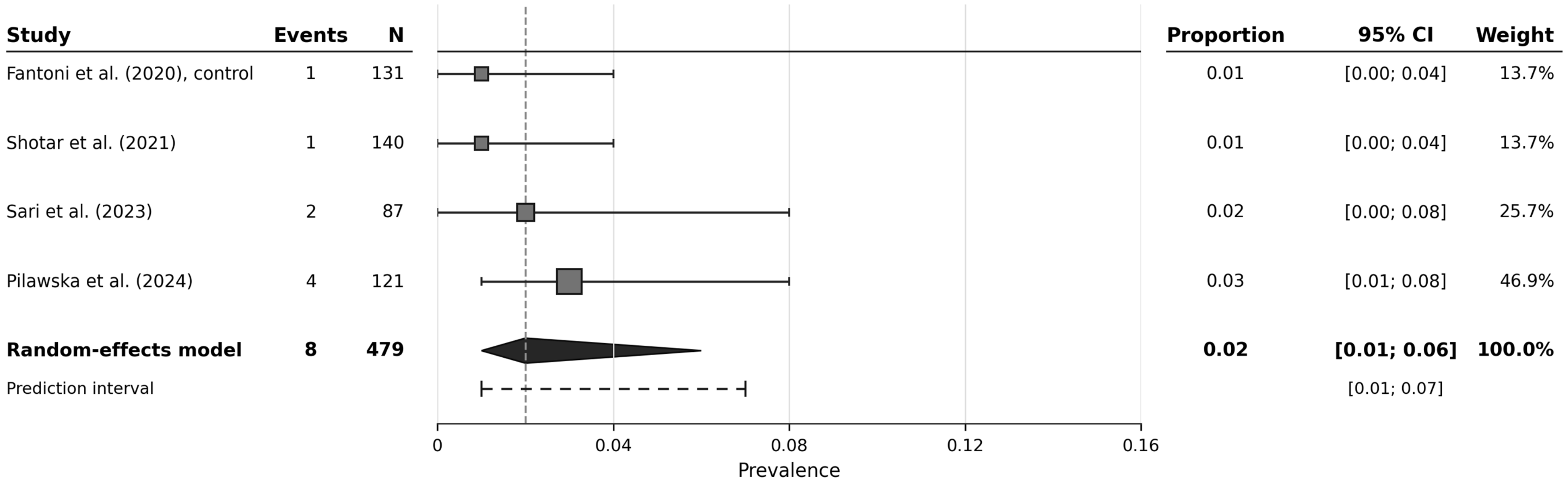

Figure S2. Sensitivity analysis for MMA arising from OA excluding the clinically selected cSDH subgroup [11-14]. Random-effects meta-analysis using logit-transformed proportions, restricted maximum likelihood estimation, and Hartung-Knapp confidence intervals.

Supplementary Table S7. PRISMA 2020 Checklist

Route-specific meningo-ophthalmic and orbitomeningeal communications relevant to middle meningeal artery embolization: a systematic review and meta-analysis

Source documents used for completion

Document	Role in checklist completion	Status
Main manuscript	Title, abstract, rationale, objectives, methods, PRISMA flow, results, figures, tables, discussion, declarations, funding, data availability, and references.	Complete
Supplementary Material	Search strategies, full-text exclusions, duplicate reports, JBI item-level appraisal, R-ready matrix, dropped/merged rows, quantitative synthesis details, and supplementary figures.	Complete
PROSPERO registration CRD420261361050	Protocol registration, planned eligibility criteria, search and screening plan, JBI appraisal, reporting bias plan, and certainty statement.	Complete
Supplementary Table S1 Search Strategies	Independent confirmation of database strings and yields; final supplement reports exact search date as 9 May 2026.	Complete

Completed PRISMA 2020 checklist

Item	Section/topic	PRISMA 2020 checklist item	Location where item is reported	Status	Completion note
**1**	Title	Identify the report as a systematic review.	Title page	Yes	The title identifies the report as a systematic review and meta-analysis.
**2**	Abstract	See the PRISMA 2020 for Abstracts checklist.	Abstract: Purpose, Methods, Results, Conclusion	Yes	The structured abstract reports rationale/objective, eligibility, registration, retrieval audit, family-specific synthesis methods, main results, interpretation, and limitations of generalizability.
**3**	Rationale	Describe the rationale for the review in the context of existing knowledge.	Introduction	Yes	The rationale is built around route-specific MMA-ophthalmic/orbitomeningeal anatomy and relevance to MMA embolization.
**4**	Objectives	Provide an explicit statement of the objective(s) or question(s) the review addresses.	End of Introduction	Yes	The objective is to synthesize MMA-ophthalmic/MMA-orbital relationships by anatomical family and pool only methodologically coherent data.

Item	Section/topic	PRISMA 2020 checklist item	Location where item is reported	Status	Completion note
**5**	Eligibility criteria	Specify the inclusion and exclusion criteria for the review and how studies were grouped for the syntheses.	Materials and Methods: Eligibility criteria; Anatomical family classification and poolability audit	Yes	Criteria specify eligible original human anatomical, angiographic and radiological evidence and define primary, exploratory, and narrative synthesis roles.
**6**	Information sources	Specify all databases, registers, websites, organisations, reference lists and other sources searched or consulted to identify studies. Specify the date when each source was last searched or consulted.	Materials and Methods: Electronic search; Supplementary Table S1	Yes	PubMed, Scopus, Web of Science, CINAHL, and LILACS are reported. Supplementary Table S1 reports the exact search date as 9 May 2026 for each database and records citation searching as n = 0 in the PRISMA flow.
**7**	Search strategy	Present the full search strategies for all databases, registers and websites, including any filters and limits used.	Supplementary Table S1	Yes	Full database-specific strategies, search dates, limits/notes, and source yields are provided.
**8**	Selection process	Specify the methods used to decide whether a study met inclusion criteria, including how many reviewers screened each record/report, whether they worked independently, and details of automation tools used.	Materials and Methods: Study selection; Results: Included articles; Supplementary Tables S2 and S2a	Yes	Deduplication, title/abstract screening, full-text retrieval, standardized exclusion reasons, and duplicate report handling are reported. Reviewer roles are supported by author contribution statements for screening.
**9**	Data collection process	Specify the methods used to collect data from reports, including how many reviewers collected data from each report, whether they worked independently, any processes for obtaining or confirming data from investigators, and any automation tools used.	Materials and Methods: Data collection process; Author contributions	Yes	Structured extraction variables and row-level audit procedures are described. Data curation and investigation roles are stated in author contributions.
**10a**	Data items	List and define all outcomes for which data were sought. Specify whether all results compatible with each outcome domain were sought and, if not, the methods used to decide which results to collect.	Materials and Methods: Data collection process; Anatomical family classification and poolability audit; Supplementary Tables S4-S6	Yes	Outcomes include anatomical family, direction/route, events, denominator, unit of analysis, evidence source, and synthesis tier.
**10b**	Data items	List and define all other variables for which data were sought. Describe any assumptions made about missing or unclear information.	Materials and Methods: Data collection process; Supplementary Tables S4 and S5	Yes	Other variables include study material/population, method, sample size, modality group, independence rationale, and dropped/merged row decisions.
**11**	Study risk of bias assessment	Specify the methods used to assess risk of bias in included studies, including details of the tool(s), how many reviewers assessed each study, whether they worked independently, and any processes for resolving disagreements.	Materials and Methods: Assessment of methodological quality; Results: Risk of bias; Table 4; Supplementary Table S3	Yes	A JBI-informed prevalence appraisal is reported. The manuscript states it informed synthesis role rather than automatic exclusion; author contributions identify risk-of-bias assessment responsibility.
**12**	Effect measures	Specify for each outcome the effect measure(s) used in the synthesis or presentation of results.	Materials and Methods: Statistical methods; Results: Table 3; Supplementary Table S6	Yes	Effect measures are proportions/pooled prevalence with 95% CI, prediction intervals, I2, tau2, and heterogeneity p-values.

Item	Section/topic	PRISMA 2020 checklist item	Location where item is reported	Status	Completion note
**13a**	Synthesis methods	Describe the processes used to decide which studies were eligible for each synthesis.	Materials and Methods: Anatomical family classification and poolability audit; Results: Table 2; Supplementary Tables S4-S5	Yes	Family-level and row-level poolability audits are explicitly described and documented.
**13b**	Synthesis methods	Describe any methods required to prepare the data for presentation or synthesis, such as handling missing summary statistics or data conversions.	Materials and Methods: Data collection process; Supplementary Tables S4-S5	Yes	Nested, duplicate, conditional, and overlapping rows were dropped or merged before synthesis; independent rows are documented.
**13c**	Synthesis methods	Describe any methods used to tabulate or visually display results of individual studies and syntheses.	Results: Tables 1-4; Figures 1-3; Supplementary Tables S4-S6; Supplementary Figures S1-S2	Yes	The manuscript and supplement use PRISMA flow, characteristics tables, poolability tables, quantitative summary tables, forest plots, and supplementary matrices.
**13d**	Synthesis methods	Describe any methods used to synthesize results and provide a rationale for model choices.	Materials and Methods: Statistical methods	Yes	No global pool was calculated; family-specific logit-transformed proportions were synthesized using REML random-effects models and Hartung-Knapp confidence intervals.
**13e**	Synthesis methods	Describe any methods used to explore possible causes of heterogeneity among study results.	Materials and Methods: Sensitivity and subgroup analyses; Results: Prevalence; Figure 3	Yes	Heterogeneity was explored descriptively through exclusion of the cSDH subgroup and evidence-source subgrouping for MLA/lacrimal-MMA.
**13f**	Synthesis methods	Describe any sensitivity analyses conducted to assess robustness of the synthesized results.	Materials and Methods: Sensitivity and subgroup analyses; Results: Prevalence; Supplementary Figure S2	Yes	Sensitivity analysis excluding the clinically selected Fantoni cSDH subgroup is reported.
**14**	Reporting bias assessment	Describe any methods used to assess risk of bias due to missing results in a synthesis.	PROSPERO; Methods and Results through retrieval audit; Supplementary Tables S2/S2a; Limitations	Yes / narrative	PROSPERO planned missing-results assessment. In the manuscript, this is addressed conservatively through retrieval audit, exclusion documentation, denominator compatibility, and non-pooling of inadequately reported families; no formal statistical reporting-bias test was appropriate for small, heterogeneous anatomical prevalence syntheses.
**15**	Certainty assessment	Describe any methods used to assess certainty or confidence in the body of evidence for each outcome.	Materials and Methods: Assessment of methodological quality; Results: Risk of bias; Limitations; PROSPERO	Not formally assessed	PROSPERO states certainty of findings would not be assessed. The manuscript explains that formal GRADE was not applied and that interpretability was addressed through JBI-informed appraisal, poolability, denominator compatibility, heterogeneity, prediction intervals, and synthesis-tier assignment.
**16a**	Study selection	Describe the results of the search and selection process, from the number of records identified to the number of studies included, ideally using a flow diagram.	Results: Included articles; Figure 1	Yes	The flow from 558 records to 19 included studies and 12 quantitative-matrix studies is fully reported.

Item	Section/topic	PRISMA 2020 checklist item	Location where item is reported	Status	Completion note
**16b**	Study selection	Cite studies that might appear to meet inclusion criteria but were excluded, and explain why they were excluded.	Supplementary Table S2; Supplementary Table S2a	Yes	Full-text excluded reports are listed with standardized reasons; duplicate reports are handled separately.
**17**	Study characteristics	Cite each included study and present its characteristics.	Results: Characteristics; Table 1; Supplementary reference key; Supplementary Tables S3-S4	Yes	The 19 included studies are cited and summarized by year, method, anatomical focus, and synthesis role.
**18**	Risk of bias in studies	Present assessments of risk of bias for each included study.	Results: Risk of bias; Table 4; Supplementary Table S3	Yes	Main manuscript summarizes JBI-informed categories; supplement provides study-level item appraisal.
**19**	Results of individual studies	For all outcomes, present for each study summary statistics and, where applicable, effect estimates and precision.	Results: Tables 2-3; Figures 2-3; Supplementary Tables S4 and S6; Supplementary Figures S1-S2	Yes	Study-level events, denominators, units, and forest plots are reported for included quantitative inputs.
**20a**	Results of syntheses	For each synthesis, briefly summarize the characteristics and risk of bias among contributing studies.	Results: Prevalence; Risk of bias; Tables 2-4; Discussion	Yes	Each synthesis is described by anatomical family, evidence source, heterogeneity, and interpretive tier.
**20b**	Results of syntheses	Present results of all statistical syntheses conducted, including summary estimate, precision, and heterogeneity.	Results: Prevalence; Table 3; Figures 2-3; Supplementary Table S6	Yes	Pooled proportions, 95% CIs, prediction intervals, I2, tau2, and heterogeneity p-values are reported.
**20c**	Results of syntheses	Present results of investigations of possible causes of heterogeneity among study results.	Results: Prevalence; Figure 3; Supplementary Table S6	Yes	MLA/lacrimal-MMA subgroup analysis by evidence source is reported descriptively.
**20d**	Results of syntheses	Present results of all sensitivity analyses conducted.	Results: Prevalence; Table 3; Supplementary Figure S2	Yes	Sensitivity analysis excluding Fantoni cSDH is reported.
**21**	Reporting biases	Present assessments of risk of bias due to missing results for each synthesis assessed.	Results, Supplementary Tables S2/S2a, Limitations	Yes / narrative	Formal statistical assessment was not performed because of small and heterogeneous family-specific syntheses; the review transparently reports retrieval losses, full-text exclusions, duplicate reports, and synthesis restrictions.
**22**	Certainty of evidence	Present assessments of certainty or confidence in the body of evidence for each outcome.	Results: Risk of bias; Discussion; Limitations	Yes	The manuscript provides an interpretive certainty framework through JBI-informed appraisal, heterogeneity, prediction intervals, and cautious synthesis-tier language.
**23a**	Discussion	Provide a general interpretation of the results in the context of other evidence.	Discussion; Clinical considerations	Yes	The discussion interprets route-specific families and quantitative findings in relation to anatomical and procedural evidence.
**23b**	Discussion	Discuss any limitations of the evidence included in the review.	Limitations	Yes	Limitations include heterogeneous design, population, detection method, unit of analysis, small samples,

Item	Section/topic	PRISMA 2020 checklist item	Location where item is reported	Status	Completion note
					historical reports, conditional denominators, and angiographic detectability.
**23c**	Discussion	Discuss any limitations of the review processes used.	Limitations; Methods; Results: Included articles	Yes	The review process limitations are addressed through retrieval audit, non-retrieval count, full-text exclusion documentation, and conservative non-pooling decisions.
**23d**	Discussion	Discuss implications of the results for practice, policy, and future research.	Clinical considerations; Discussion; Conclusions	Yes	Implications include route-specific angiographic evaluation, standardized reporting of directionality/denominators, and cautious procedural interpretation.
**24a**	Registration and protocol	Provide registration information for the review, including register name and registration number, or state that the review was not registered.	Abstract; Materials and Methods: Protocol and registration	Yes	PROSPERO registration CRD420261361050 is reported.
**24b**	Registration and protocol	Indicate where the review protocol can be accessed, or state that a protocol was not prepared.	PROSPERO record; Materials and Methods: Protocol and registration	Yes	The PROSPERO registration is available under CRD420261361050; the manuscript refers to registration and the Supplementary Material for amendment handling.
**24c**	Registration and protocol	Describe and explain any amendments to information provided at registration or in the protocol.	Materials and Methods: Protocol and registration; Supplementary Material; PROSPERO	Yes	The amendment clarifies handling of selected pediatric, mixed-age, and cadaveric anatomical evidence for anatomical mapping and exploratory synthesis.
**25**	Support	Describe sources of financial or non-financial support for the review and the role of funders or sponsors.	Funding	Yes	The manuscript states that the research received no external funding.
**26**	Competing interests	Declare any competing interests of review authors.	Declarations: Conflict of interest	Yes	The manuscript states that the authors declare no conflicts of interest.
**27**	Availability of data, code and other materials	Report which data, code, and other materials are publicly available and where they can be found.	Data availability; Supplementary Material; Supplementary Tables S4-S6	Yes	All qualitative and quantitative data are provided in the manuscript and Supplementary Material; extraction and R-ready matrices are provided; analysis code is available from the corresponding author upon reasonable request.
